# Pathogenic Potential and Antibiotic Susceptibility: A Comprehensive Study of Enterococci from Different Ecological Settings

**DOI:** 10.3390/pathogens13010036

**Published:** 2023-12-29

**Authors:** Maria Pandova, Yoana Kizheva, Margarita Tsenova, Mariya Rusinova, Tsvetomira Borisova, Petya Hristova

**Affiliations:** 1 Department of General and Industrial Microbiology, Faculty of Biology, Sofia University, 1164 Sofia, Bulgaria; maria.pandova@abv.bg (M.P.); mhcenova@biofac.uni-sofia.bg (M.T.); pkabad@biofac.uni-sofia.bg (P.H.); 2 Human Milk Bank Bulgaria, 1309 Sofia, Bulgaria; bmk@milkbank.bg (M.R.); cv_borisova@abv.bg (T.B.)

**Keywords:** enterococcus, pathogenic potential, virulence factors, antibiotic resistance, alimentary chain

## Abstract

The pathway and the lifestyle of known enterococcus species are too complicated. The aim of the present study is to trace the path of pathogenicity of enterococci isolated from seven habitats (*Cornu aspersum* intestine; Bulgarian yoghurt; goat and cow feta cheese—mature and young, respectively; Arabian street food—doner kebab; cow milk; and human breast milk) by comparing their pathogenic potential. In total, 72 enterococcal strains were isolated and identified by MALDI-TOF, sequencing, and PCR. Hemolytic and gelatinase activity were biochemically determined. PCR was carried out for detection of virulence factors (*cylB*, *esp*, *gls24*, *nucl*, *psaA*, *agg, gelE*, and *ace*) and antibiotic resistance (*erm*, *ermB*, *blaZ*, *vanA*, *aphA*, *mefA*, *gyrA*, *cat_pIP501_*, and *aac6′-aph2″*). Phenotypic antibiotic resistance was assigned according to EUCAST. Eleven representatives of the genus *Enterococcus* were identified: *E. mundtii*, *E. casseliflavus*, *E. gilvus*, *E. pseudoavium*, *E. pallens*, *E. malodoratus*, *E. devriesei*, *E. gallinarum*, *E. durans*, *E. faecium*, and *E. faecalis.* Twenty-two strains expressed α-hemolysis. Thirteen strains had the *cylB* gene. Only two strains expressed α-hemolysis and possessed the *cylB* gene simultaneously. Positive amplification for *gelE* was found in 35% of the isolates, but phenotypic gelatinase activity was observed only in three strains. All isolates showed varying antibiotic resistance. Only *E. faecalis* BM15 showed multiple resistance (AMP-HLSR-RP). Correlation between genotypic and phenotypic macrolide resistance was revealed for two *E. faecalis* strains.

## 1. Introduction

The members of the genus *Enterococcus* are bacteria that have a dual role in the environment: positive (as commensal and potential probiotic bacteria) and negative (opportunistic pathogens capable of infecting plants, animals, and humans) [1]. The pathway and the lifestyle of known enterococcus species in the natural environment are far too complicated and poorly studied. Most studies characterize enterococci isolated from particular ecological niches and do not track what features they develop when they jump from one biological kingdom to another.

Enterococci are ubiquitous Gram-positive bacteria that can be found in various ecological niches, such as environmental, clinical, and food. This genus of bacteria forms a part of natural biomes of soil, water, sewage, and arable land, as well as populations in the gastrointestinal tracts (GITs) of mammals, birds, fishes, invertebrates, and insects [2,3,4,5,6]. Similarly, enterococci have been isolated from fresh vegetables (olive, pepper, celery, cilantro, mustard greens, spinach, collards, parsley, dill, cabbage, and cantaloupe) and wild plants also [7,8,9,10]. Flowering plants and crops have also been known to be carriers of enterococci [11]. Mundt [10] states that relationships between enterococci and plants are based mainly on their epiphytic persistence. However, they have been considered temporary inhabitants as a result of wind and insect activity [10].

Enterococci are a diverse taxonomic group that includes 58 recognized species and 2 subspecies [12]. The most distributed members of the genus in the GITs of mammals have been reported to be *Enterococcus faecalis*, *Enterococcus faecium*, *Enterococcus durans*, and *Enterococcus hirae* [13]. Plant-associated epiphytic *Enterococcus* species most commonly belong to *E. faecalis*, *Enterococcus mundtii*, *Enterococcus casseliflavus*, *E. faecium*, and *Enterococcus sulfureus* [11,14,15]. A new taxonomic species, *Enterococcus plantarum* sp. nov., was identified during the study of the microflora of various plants from meadows [16]. However, most studies assume that enterococci, as part of the natural microbiota of the gastrointestinal tract of warm-blooded animals, can enter the environment through feces, contaminate soil and water, and then colonize plants. This pathway explains why enterococci predominate on plant surfaces and are resistant to a number of antibiotics [17,18,19,20], but at the same time, they can be identified as potential cross-over agents promoting the dissemination of antibiotic resistance [8,21]. Furthermore, it has been suggested that the infection strategies of some enterococci (*E. faecalis*) are similar in plants, mammals, and nematodes [1].

Moreover, evidence that some strains of *E. faecalis* can infect the roots and leaves of the plant *Arabidopsis thaliana*, causing local and systemic infection that leads to the death of the infected plant, has been reported [1]. *E. faecalis* has developed a significant biofilm-like pathogenic community that has colonized the root surface [1]. Enterococci have been previously reported as a component of the microbiome of pepper plants with symptoms of disease [8].

On the other hand, it can be assumed that enterococci are part of the plant microbiome and that they enter the intestinal tracts of animals and humans through the intake of plant food. Each gut microbiome selects the enterococcal species it needs to maintain eubiosis. Colonizing different microbiomes, from invertebrates to mammals, allows plant enterococci to acquire new genes, which they then spread into new environmental niches. This life cycle of passing through hosts from different biological kingdoms defines enterococci as important vectors for the horizontal transfer of antibiotic resistance and virulence genes, despite where they originate from [22].

Traditionally, enterococci have been considered to be normal commensal bacteria and may even be beneficial for a variety of gastrointestinal and systemic illnesses. Some enterococci species have the ability to stimulate the immune system and play an important role in the maintenance of intestinal homeostasis [23,24]. Similarly, enterococci have an active part in food technology as the starting culture in meat and cheese fermentation [25], as well as in food preservation [26,27,28].

However, enterococci can cause invasive infections if their relationship with the host is broken [29]. These bacteria exhibit remarkable adaptability in colonizing different hosts and show the ability to thrive as pathogens in diverse ecological niches [30]. However, some strains have acquired a wide range of virulence and antibiotic resistance genes, leading to an increase in their pathogenicity and posing a significant public health challenge [31,32]. Thus, enterococci, despite their commensal nature, have been identified as the most prevalent causes of urinary tract infections and nosocomial bacteremia. They also constitute the second most commonly reported cause of surgical wound infections and the third most often reported cause of bacteremia [33,34]. Moreover, enterococci have been reported as the main Gram-positive bacteria causing hospital-acquired infections during and after the COVID 19 pandemic [35,36,37,38,39,40].

The virulence factors that contribute to enterococcal pathogenesis include collagen-binding protein (Ace), aggregation substance (Agg), surface proteins (Esp), cytolysin (Cyl), gelatinase (Gel), general stress protein (Gls24), and immune evasion molecules [41]. Defined as effector molecules, virulence factors indicate a high potential of enterococci for host adherence, tissue invasion, immune evacuation, and nutrient acquisition. Ace is an adhesin, anchored to the cell wall, that helps enterococcal species to adhere to collagen. The agglutination substance (Agg) is a pheromone-inducible surface protein which helps in aggregation during the conjugation process. Cytolysin (Cyl) production is associated with the capacity of bacteria to access the bloodstream and trigger septicemia. Epidemiological research has found that the enterococcal surface protein, Esp, is typically linked with infectious strains, compared to commensal isolates, and is located on a large pathogenicity island [42]. Esp is also involved in initial adherence and biofilm formation and contributes to the pathogenesis of different infections. Gelatinase (Gel) is a zinc metallo-endopeptidase which takes part in pathogenesis by making nutrients available through degradation of host tissue and by taking part in biofilm formation [43].

A notable feature of enterococci is their intrinsic resistance to cephalosporin, cotrimoxazole, lincomycin, and low levels of penicillin and aminoglycosides. Enterococci can also acquire resistance genes from other microorganisms through horizontal gene transfer and thus become resistant to a variety of antibiotics such as chloramphenicol, tetracycline, streptogramin, macrolides, a high level of glycopeptide, aminoglycosides, and quinolones [44]. This acquired resistance along with their known remarkable ability to overcome and adapt to various environmental stress factors, give the enterococci the unique potential to realize complex lifestyles [45].

Therefore, the study of the diversity and distribution of pathogenicity-determining genes of enterococcal populations of different origins provides valuable insight into their adaptive strategies in different hosts and environments. It is also critical to understand the pathogenicity mechanisms that these multi-host pathogens possess. Moreover, the comparison of the virulence and resistance arsenal of the enterococcal populations, adapted to inhabit completely different niches, contributes to the global knowledge of enterococcal lifestyle and reveals the key role of the evolutionary pressure of the habitat on it. The present study considers enterococcal populations from different biological kingdoms/origins as a reservoir of genes for virulence and antibiotic resistance with respect to possible re-return into the environment and subsequent colonization of plants or other diverse ecological niches such as soil and water. The aim is to compare the pathogenic potential of enterococci isolated from herbivorous invertebrate animals, food products derived from herbivorous warm-blooded animals, and human breast milk.

## 2. Materials and Methods

### 2.1. Sample Collection and Isolation of the Bacteria

In total, twenty-seven samples from animal GIT (invertebrate herbivorous species *Cornu aspersum* at the hibernation stage of the life cycle) and food (Bulgarian yoghurt; goat and cow feta cheese—mature and young, respectively; Arabian street food—doner kebab; cow milk; and human breast milk) were used for *Enterococcus* species isolation. Breast milk samples were supplied by the Human Milk Bank, Bulgaria, *C. aspersum* samples were collected and processed according to Koleva et al. [46], and food samples were obtained randomly from artisanal markets. Approximately 1 g or 1 mL from each sample was homogenized in saline (at a ratio of 1:9) and all samples were directly cultivated on the selective medium Slanetz and Bartley agar (HiMedia Laboratories, Mumbai, India). The plates were cultivated at 37 °C for 24 h–48 h. The appearance of dark red-brownish colonies on the surface of the used media after the cultivation served as positive results for selection of enterococcal strains. Pure cultures from separate colonies were isolated as potential *Enterococcus* species after double purification. Three reference strains were also used in this study: *E. faecalis* NBIMCC 3915 and *E. faecium* NBIMCC 8754 as positive controls for the genus and species PCR identification, and *Bacillus cereus* NBIMCC 1085 as positive control for β-hemolytic activity in the hemolysis assay.

### 2.2. DNA Preparation

The bacterial cultures were cultivated in MRS broth (HiMedia, Mumbai, India) at 37 °C for 24 h prior to the genomic DNA extraction. The biomass was harvested by centrifugation at 10,000× *g* and was washed twice with 500 µL 1% NaCl. Total DNA was extracted by E.Z.N.A. Bacterial DNA Kit (Omega Biotek Inc., 400 Pinnacte Way, Suite 450, Norcross, GA, USA). For improved lysis of the cells, 2 µL 1000 units/mg mutanolysin (Merck KGaA, Darmstadt, Germany) was added at the enzyme lysis step.

### 2.3. Species Identification

The isolates were identified by three different methods: PCR with genus- and species-specific primers [46,47], 16S rRNA sequencing [46], and MALDI-TOF (Matrix-Assisted Laser Desorption/Ionization Time-of-Flight) mass spectrometry [8]. Genus- and species-specific PCRs were performed in a total reaction volume of 25 µL containing 16.5 µL ultrapure H_2_O, 0.5 µL (5 pmol/µL) of each primer, 6.5 µL VWR Red Taq polymerase master Mix (VWR International bvba/sprl, Haasrode Researchpark Zone 3, Geldenaaksebaan 464 B-3001, Haasrode Belgium), and 1 µL extracted DNA. The reactions conditions were as follows: initial denaturation at 95 °C for 5 min, followed by 25 cycles of denaturation at 94 °C for 45 s, annealing at 58 °C, 50 °C, 60 °C, and 55 °C, according to primer specificity [46,47] for 45 s, extension step at 72 °C for 45 s, and a final extension step at 72 °C for 7 min. PCR products were separated in 1.5% agarose gel electrophoresis at 100 V for 30 min, stained with ethidium bromide, and visualized under UV light. Molecular size marker 100 bp DNA ladder (SERVA FastLoad 100 bp DNA ladder, SERVA Electrophoresis GmbH, Carl-Benz-Str. 7, Heidelberg, Germany) was used. The universal primers 9F and 1542R were used to amplify the 16S rRNA gene [48]. Purified PCR products were sequenced in Macrogen Europe, Meibergdreef 57 1105 BA, Amsterdam, The Netherlands. The obtained sequences were subjected to comparative analyses using nucleotide BLAST (NCBI, accessed on June 2021).

### 2.4. Phenotypic Hemolytic Activity Assay

The evaluation of hemolytic activity was performed according to the method described by Carrillo et al. [49]. Pure bacterial cultures were cultivated overnight on brain heart infusion (BHI) agar (HiMedia Inc., Mumbai, India) to obtain log-phase cultures. Then, the cultures were surface spot inoculated on Columbia agar plates supplemented with 5% horse blood and incubated at a temperature of 37 °C for a duration of 24 to 48 h, after which the plates were examined for hemolysis. Clear zones around the colonies were interpreted as β-hemolysis (positive) and lack of zone was reported as gamma-hemolysis (negative). When greenish zones were observed, the strains were reported as α-hemolytic and taken as negative for the assessment of β-hemolytic activity [50].

### 2.5. Phenotypic Gelatinase Activity Assay

The evaluation of phenotypic gelatinase activity was carried out according to the procedure described by [41]. Pure bacterial cultures were cultivated overnight on BHI agar (HiMedia Inc., India) to obtain log-phase cultures. Then, the cultures were surface spot inoculated on agar plates containing 5 g/L peptone (Merck, Darmstadt, Germany), 30 g/L gelatin (Difco, Detroit, MI, USA), 3 g/L yeast extract (Gibco, Paisley, Scotland), and 15 g/L agar (Plant agar, Duchefa Biochemie, The Netherlands), with a pH of 7.0, and were incubated at 37 °C for 48 h. After the cultivation, the agar surface was flooded with a saturated solution of (NH_3_)_2_SO_4_ (55 g/100 mL dH_2_O). Gelatinase producers formed clear zones around the spots, and these results were interpreted as positive.

### 2.6. Antibiotic Susceptibility Testing

Susceptibility to antibiotic substances was performed using the Kirby–Bauer disc diffusion method [51]. For evaluation of antibiotic resistance of enterococcal isolates, fifteen antibiotics were tested: ampicillin 2 μg/disc (AMP), imipenem 10 µg/disc (IPM), ciprofloxacin 5 µg/disc (CP), levofloxacin 5 µg/disc (LE), norfloxacin 10 µg/disc (NX), gentamicin 30 µg/disc—test for high-level aminoglycoside resistance (GEN), streptomycin 300 µg/disc—test for high-level streptomycin resistance (HLS), teicoplanin 30 µg/disc (TEI), vancomycin 5 µg/disc (VA), quinupristin-dalfopristin 15 µg/disc (RP), eravacycline 20 µg/disc (ERV), tigecycline 15 µg/disc (TG), linezolid 10 µg/disc (LZ), nitrofurantoin 100 µg/disc (NIT), and trimethoprim 5 µg/disc (TR). The whole procedure of testing of the antibiotic susceptibility along with the interpretation of the obtained results was carried out according to European Committee on Antimicrobial Susceptibility Testing guidelines [52].

### 2.7. PCR Amplification of Virulence and Antibiotic Resistance Genes

PCR was carried out for the detection of eight virulence (*cylB*, *esp*, *gls24*, *nucl*, *psaA*, *agg, gelE*, and *ace*) and nine antibiotic resistance-related genes (*erm*, *ermB*, *blaZ*, *vanA*, *aphA*, *mefA*, *gyrA*, *cat_pIP501_*, and *aac6′-aph2″*) commonly presented in clinical and environmental enterococci. PCR mixtures were prepared as described above (see Section 2.3). The reaction conditions were as follows: initial denaturation at 95 °C for 5 min, followed by 25 cycles of denaturation at 94 °C for 45 s, annealing temperature according to primer specificity (Table 1) for 45 s, extension step at 72 °C for 45 s, and a final extension step at 72 °C for 7 min. PCR products were visualized in a 1.5% agarose gel electrophoresis at 100 V for 30 min.

### 2.8. Data Analysis

Welch’s *t*-test was used to compare the number of resistance and virulence genes, as well as the number of phenotypic antibiotic resistance profiles of isolates from different origins and within different species. Results were considered significant when *p* < 0.05.

## 3. Results

### 3.1. Bacterial Isolation and Identification

**Figure 1:**
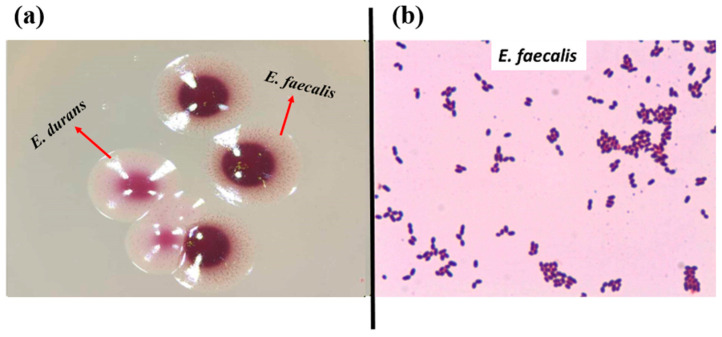
(**a**) Strains YFC1 (*E. faecalis*) and YFC2 (*E. durans*) on Slanetz and Bartley medium; (**b**) Gram staining of strain YFC3 (*E. faecalis* from young feta cheese).

In total, 72 presumptive enterococcal strains were isolated from various ecological niches. Seventeen strains were isolated from the GIT of *C. aspersum* at the hibernation stage of the life cycle, as described previously [46]. Thirty-nine strains were isolated from different food sources (27 from Bulgarian yogurt, 2 from matured goat feta cheese, 5 from young cow feta cheese, 1 from doner kebab, and 4 from cow milk). Sixteen strains were isolated from human breast milk. The latest strains were grouped as human enterococci with non-hospital origin. All isolates appeared as pink or dark red-brownish colonies when streaked on the selective Slanetz and Bartley medium (Figure 1a). Under the microscope, they were Gram-positive cocci or coccobacilli, grouped in clusters, chains, or pairs (Figure 1b).

Three different approaches were used for species identification: PCR with genus- and species-specific primers, 16S rRNA sequencing, and MALDI-TOF mass spectrometry. The comparative analyses of the obtained sequencing results showed similarity percentage above 98–99%, which is considered a very good species identification. All obtained results for the species identification with MALDI-TOF showed score values above 2.0, which represents reliable species-level identification. Detailed information for the species identification is given in Table 2.

**Figure 2:**
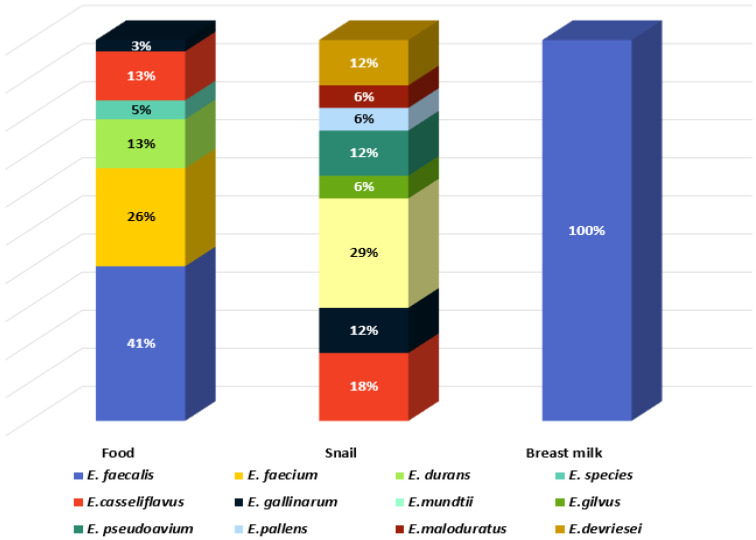
Species diversity in the analyzed habitats.

Eleven species were identified: *E. mundtii*, *E. casseliflavus*, *Enterococcus gilvus*, *Enterococcus pseudoavium*, *Enterococcus pallens*, *Enterococcus malodoratus*, *Enterococcus devriesei*, *Enterococcus gallinarum*, *E. durans*, *E. faecium*, and *E. faecalis*. Two isolates were identified at genus level as *Enterococcus* spp. (BY7 and BY8, isolated from Bulgarian yoghurt). The greatest species diversity was established in the GIT of *C. aspersum* as eight species were identified: 29% *E. mundtii*, 18% *E. casseliflavus*, 6% *E. gilvus*, 12% *E. pseudoavium*, 6% *E. pallens*, 6% *E. malodoratus*, 12% *E. devriesei*, and 12% *E. gallinarum* (Figure 2).

Two of the species (*E. mundtii* and *E. casseliflavus*), generally recognized as plant-associated enterococci [15], represent 47% of the enterococcal population of the GIT of the snail, which is a herbivore. *E. faecalis* and *E. faecium* were not detected in the GIT of *C. aspersum*. In contrast, these two species were predominantly identified in the food samples: 41% and 26%, respectively. The species *E. durans* (6%) was isolated from cow milk and young feta cheese from cow milk. All isolates from human breast milk were identified as *E. faecalis.*

### 3.2. Occurrence of cylB Gene and Production of Hemolysin

**Figure 3:**
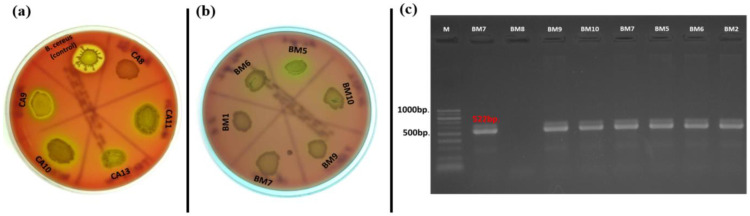
(**a**) Hemolysis test on Columbia agar plate supplemented with 5% horse blood; (**b**) α-hemolysis of *E. faecalis* isolated from breast milk; (**c**) Gel electrophoresis of PCR amplification products for the *cylB* gene of breast milk isolates.

Hemolytic activity of the *Enterococcus* species is considered one of the basic virulence factors influencing their pathogenicity. Our results showed that there were no strains that showed phenotypic β-hemolytic activity on Columbia agar + 5% horse blood, but some strains expressed α-hemolysis (31% of all tested strains) (Figure 3a). Of the 17 strains isolated from *C. aspersum* GIT, 10 representatives of the species *E. casseliflavus* (*n* = 2), *E. gilvus* (*n* = 1), *E. gallinarum* (*n* = 2), *E. pseudoavium* (*n* = 1), *E. pallens* (*n* = 1), *E. malodoratus* (*n* = 1), and *E. devriesei* (*n* = 2) showed phenotypic α-hemolytic activity. The only species in this group not showing hemolytic activity was the plant-associated species *E. mundtii*. Of the 39 strains isolated from food samples (cow milk, Bulgarian yogurt, young feta cheese, and mature feta cheese), 11 were α-hemolytic. However, such activity was observed among *E. durans* (YFC2), *E. casseliflavus* (BY19), *E. gallinarum* (BY17), *Enterococcus* sp. (BY8), *E. faecalis* (*n* = 4), and *E. faecium* (*n* = 3). Surprisingly, only one strain isolated from human breast milk possessed α-hemolytic activity (*E. faecalis* BM5). Of great importance was the correlation between phenotypic hemolytic expression and the related genotypic determinants. The *cylB* gene is a member of the *cyl* operon, responsible for the synthesis of cytolysin and for β-hemolytic activity, respectively [62]. Thirteen out of all the tested strains had the *cylB* gene (9 from human breast milk and 4 from food samples), but none of them expressed β-hemolytic activity. Only two strains (*E. faecium* DK1 and *E. faecalis* BM5) expressed α-hemolysis and possessed the *cylB* gene simultaneously (Figure 3 b,c). None of the strains isolated from the GIT of the snail possessed the *cylB* gene.

### 3.3. Occurrence of gelE and Production of Gelatinase

**Figure 4:**
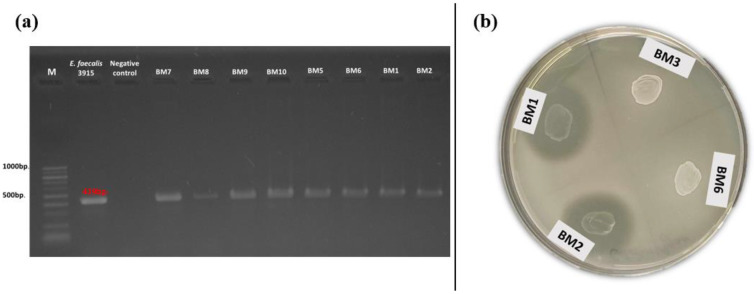
(**a**) Gel electrophoresis of PCR amplification products for the *gelE* gene of breast milk isolates; (**b**) Phenotypic gelatinase activity of breast milk isolates (BM1 and BM2) on gelatin agar.

The production of gelatinase and the occurrence of the related gene (*gelE*) were also investigated. Positive amplification for *gelE* was found in 35% of the tested isolates (Figure 4a). Of these, 11 had a food origin and 14 were isolated from breast milk. All of them belonged to the species *E. faecalis* and *E. faecium*. Simultaneous occurrence of phenotypic gelatinase activity and the related genotypic determinant (*gelE*) was observed only in three enterococcal strains (*E. faecalis* BM1, BM2, and BM11) isolated from human breast milk (Figure 4b). None of the snail isolates had the abovementioned gene.

### 3.4. Phenotypic Antibiotic Resistance

The obtained results from the phenotypic antibiotic resistance were interpreted according to EUCAST, 2019 [63]. All isolates were susceptible to fluoroquinolone antibiotics (ciprofloxacin, levofloxacin, and norfloxacin), teicoplanin, linezolid, nitrofurantoin, vancomycin, and imipenem. Resistance to ampicillin was observed in 21% (n = 15) of all tested strains (Table 3). Among them, 53% were *E. faecalis* isolated from human breast milk.

**Figure 5:**
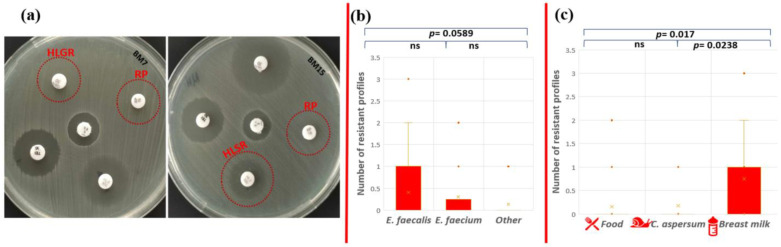
Phenotypic antibiotic resistance test. (**a**) Red circles indicate HLGR and RP resistance of *E. faecalis* BM7 and HLSR (colonies within the zone) and RP resistance of *E. faecalis* BM15; statistical analyses include comparison of the number of ABR profiles (**b**) between the different enterococcal species and (**c**) between the different strain origins. Significant difference was considered *p* < 0.05; ns corresponds to non-significant difference.

High-level gentamicin resistance (HLGR), high-level streptomycin resistance (HLSR), and quinupristin-dalfopristin resistance (RP) were established for two isolates with human origin (Figure 5a).

The only strain that showed multidrug phenotypic resistance profile to three antibiotics (AMP-HLSR-RP) was *E. faecalis* BM15 isolated from human breast milk (1.38% from all tested strains). Phenotypic resistance to two antibiotics was observed for strains *E. faecium* CM1 (AMP-ERV), *E. faecalis* YFC1 (AMP-TG), and *E. faecalis* BM7 (HLGR-RP). However, the statistical analysis showed that there is no significant difference in the number of resistance profiles between *E. faecalis* and *E. faecium* (*p* = 0.674); *E. faecalis* and other *Enterococcus* species (*p* = 0.0589); and *E. faecium* and other *Enterococcus* species (*p* = 0.471) (Figure 5b). The human isolates (breast milk) exhibited patterns of resistance to more antibiotics compared to the other two groups (food and snail isolates). A significant difference between antibiotic resistance phenotype profiles was established between strains from food and breast milk (*p* = 0.017), as well as strains from snail and breast milk (*p* = 0.0238), but not between isolates from snail and food (*p* = 0.855) (Figure 5c).

### 3.5. Screening for Antibiotic Resistance Genes

**Figure 6:**
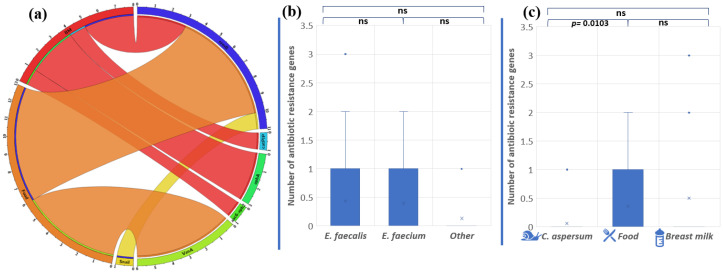
(**a**) Distribution of antibiotic resistance genes in enterococci from food, snail, and breast milk (labeled BM in the circus plot) (the image was generated with Circos Table Viewer v0.63-10). The outer ring of the circus plot represents the number of isolates that carry the analyzed genes. Connecting lines between the specific genes and the origin of the isolates are shown if the gene was detected in isolates from any of the three origins. Comparison of the number of antibiotic resistance genes between the different enterococcal species (**b**) and the different strains’ origins (**c**). Significant difference is considered *p* < 0.05.

Overall, the abundance of antibiotic resistance genes in the analyzed strains was low. Only 16 (22%) of all isolates showed the presence of one or more antibiotic resistance genes (Figure 6a).

The gene *ermB*, associated with macrolide resistance, was most frequently found among the analyzed *Enterococcus* population (15, 2%), followed by *vanA* (8, 3%), *aphA3* (4, 2%), *aac6′-aph2″* (1, 4%), and *cat_pIP501_* (1, 4%). We established that two *E. faecalis* strains (BM15 and BM7) had three genes encoding antibiotic resistance, which makes them unsusceptible to macrolide and aminoglycoside antibiotics. Strain BM7 showed the presence of *ermB*, *aphA3*, and *aac6′-aph2″*. Strain BM15 possesses the genes *ermB*, *cat_pIP501_*, and *aphA3*. These data showed a correlation between genotypic and phenotypic antibiotic resistance to macrolides. However, the other gene responsible for macrolide resistance (*mefA)* was not detected. Unexpectedly, the gene *vanA*, associated with vancomycin resistance, was found in six of our strains: five *E. faecalis* strains from Bulgarian yogurt (BY2, BY3, BY4, BY5, BY6) and one *E. faecium* strain from mature feta cheese (MFC1), although no phenotypic appearance was observed.

The species comparison showed no significant differences in the number of antibiotic resistance genes (*p* = 0.8897 between *E. faecalis* and *E. faecium*; *p* = 0.0665 between *E. faecalis* and other *Enterococcus* species; and *p* = 0.273 between *E. faecium* and other *Enterococcus* species) (Figure 6b). On the other hand, the origin comparison showed significant differences between the snail and food distribution of antibiotic resistance genes (*p* = 0.0103). The above were not observed between food and breast milk isolates (*p* = 0.569) or between snail and breast milk isolates (*p* = 0.135) (Figure 6c).

Only one strain (BM7), having the HLGR gene *aac6′-aph2″*, showed the relevant phenotypic resistance to 30 µg/disc gentamicin. Fifteen of the isolates (20%) showed phenotypic resistance to ampicillin, but none of the strains had *blaZ* (codes β-lactamases) in its genome. The gene *gyrA* was also absent and, as expected, resistance to fluoroquinolones (ciprofloxacin, norfloxacin, and levofloxacin) was not observed.

### 3.6. Screening for Virulence-Associated Genes

**Figure 7:**
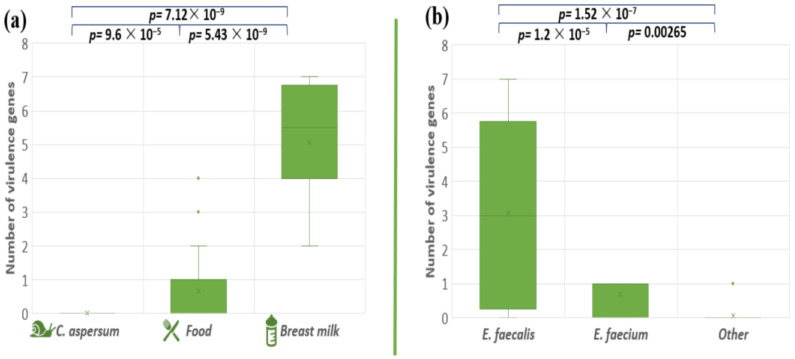
Comparison of the number of virulence genes between the different enterococcal species (**a**) and the different strains’ origins (**b**). Significant difference is considered *p* < 0.05.

The pathogenicity degree of the pathogenic microorganisms depends on genetically determined virulence factors. The presence of a total of eight virulence genes (*cylB*, *esp*, *gls24*, *nucl*, *psaA*, *agg*, *gelE*, and *ace*) among our enterococcal isolates was investigated (Table 4). The analyses of the distribution of the tested virulence-associated genes showed that the snail isolates did not possess any of the analyzed virulence genes. Stress protein regulator (*gls24-like*) was not found in the investigated enterococcal isolates. The most amplified gene among all the isolates was the gelatinase gene (*gelE*), followed by the Mn-transporter *psaA*: 31% and 28%, respectively. Both genes responsible for the synthesis of enterococcal surface protein (*esp*) and nuclease (*nucl*) were presented in 19.4% of all tested strains. Genes responsible for hemolytic activity (*cylB*) and collagen-binding protein (*ace*) were detected in 18% of the enterococcal population. Genetic determinants for aggregation substance (Agg) were found in 15.2% of the tested isolates. Four human breast milk isolates (BM5, BM6, BM9, and BM10) contain seven out of the eight screened virulence genes. The distribution of all tested virulence factors among food isolates were strain specific. The comparison of the distribution of the virulence factors in enterococcal strains isolated from the different ecological niches, showed significant differences (between snail and food isolates *p* = 9.6 × 10^−5^; food and breast milk *p* = 5.43 × 10^−9^; snail and breast milk *p* = 7.12 × 10^−9^) (Figure 7a). A similar tendency was observed between *E. faecalis* and *E. faecium p* = 1.2 × 10^−5^; *E. faecium* and other *Enterococcus* species *p* = 0.00265; *E. faecalis* and other *Enterococcus* species *p* = 1.52 × 10^−7^ (Figure 7b).

## 4. Discussion

The multi-host lifestyle and unique adaptability of enterococci lead to interconnected microbiomes between mammals, invertebrates, insects, and plants which facilitate the acquisition and spread of virulence and antibiotic resistance genes (ABR) [64]. Therefore, the enterococcal populations from different biological kingdoms/origins represent reservoirs of factors causing infections in humans and plants [1,65]. In this study, we compared the pathogenic potential of enterococci isolated from diverse habitats with respect to assess their possible virulent potential for subsequent colonization of plants after potential re-return into the environment.

Our main hypothesis was that, in passing through hosts from different kingdoms, enterococci successfully adapt to the current habitat by acquiring various virulence and ABR genes, which helps them in interspecies relationships. Therefore, it is mandatory to investigate in depth the pathogenic potential of enterococci originating from various ecological niches. This accumulated knowledge could be useful in evaluating the potential risk of undesired genetic burden in the environment after the eventual re-entering of the enterococci (with acquired virulence and ABR potential) into the environment.

As a primary source of food for many organisms, plants, along with soil and water, can act as reservoirs for enterococcal species, which can subsequently join the path of pathogenicity and be transmitted through the animal chain mainly by herbivorous animals. A good example is the *C. aspersum* species of snail, that is in touch with all these habitats and can itself be used as a food source for other animals, including humans. By studying the microbiome of the snail, the microbial presence in its food (plants) can be deducted.

In our study, in total, 72 enterococcal strains, representatives of 11 species and isolated from seven habitats, were characterized (Table 2). In this study, we found eight enterococci species in the snail intestinal tract, with *E. mundtii* and *E. casseliflavus* being the most prevalent. *E. casseliflavus* was also established in Bulgarian yoghurt (fermented cow milk), derived from herbivorous warm-blooded animal (cow), but not in our isolates from raw cow milk itself. However, the persistence of *E. casseliflavus* in raw bovine milk has been reported [66]. Surprisingly, in the GIT of the snail, none of the isolates belonged to the *E. faecium* or *E. faecalis* species but both species dominated in all other samples. The other six species found in the GIT of *C. aspersum* have been generally reported to have human and animal origins [15]. It can be suggested that these species of bacteria have moved into the plants from soil and water and from there into the GIT of the snail [21]. Our results showed that the potential plant-associated isolates in the GIT and food (*E. casseliflavus*) did not carry genes for virulence and antibiotic resistance. Only three isolates (*E. mundtii* CA1, *E. malodoratus* CA11, and *E. devriesei* CA13) were phenotypically resistant to ampicillin. However, a study found that enterococci isolated from raw and processed plant-derived foods have a quite different phenotypic and molecular profile of antibiotic resistance [21]. The authors of the study found that *E. faecium*, *E. faecalis*, and *E. casseliflavus* strains are resistant to erythromycin, streptomycin, tigecycline, fosfomycin, and rifampicin but not to ampicillin. In that study, correlation between phenotypic high aminoglycoside resistance (HLAR) and the related genetic determinants (*ant(6′)-Ia, aph(3′)-IIIa and aac(6′)-Ie-aph(2″)-la*) has been reported [21]. An interesting result was that the species *E. gallinarum* was found in two of our samples: snail GIT and Bulgarian yoghurt. We established that no virulence or ABR was found in the snail isolates (*E. gallinarum* CA14 and CA15), as opposed to the Bulgarian yoghurt isolate (*E. gallinarum* BY17), which was found to carry the gene for ampicillin resistance. Thus, we can conclude that enterococci from snails and their food source, namely plants, did not represent any threat to human health. The acquisition of ampicillin resistance may likely happen in some of the later stages of the alimentary chain.

An important reservoir for the dissemination of enterococcal populations is the products of the lactation of mammals. For example, the most commonly isolated species from goat and sheep raw milk and their products (cheese) have been reported to be *E. faecalis* and *E. faecium* [67]. In our investigation, two similar milk products were analyzed—from cows and humans. To our knowledge, the enterococcal population in human breast milk is poorly studied. Breast milk has complex nutrient composition and contains a variety of bacterial species which influence infant health and immunity [68]. Some authors have even suggested that the enterococcal abundance corelates with the infants’ excessive weight gain [69]. The species *E. faecalis*, *E. faecium*, *E. hirae*, *E. casseliflavus*, and *E. durans* have been reported to be found in the milk of healthy women [69,70,71]. In our cow milk samples, we found three species—*E. faecalis*, *E. faecium*, and *E. durans*, which is in accordance with other authors’ findings [67]. According to some authors, of all reported plant-associated enterococcal species, only *E. faecalis*, *E. faecium*, and *E. casseliflavus* are dominant and best adapted to mammals [69]. It has been suggested that this selection is due to the extreme genomic plasticity of these species, allowing for facile horizontal gene transfer [45]. However, in our study, only strains of *E. faecalis* were identified in human breast milk. Our results showed that the distribution of the virulence and ABR genes was greatest among the enterococcal population in this ecological niche. It has been reported that *E. faecalis* and *E. faecium* have the greatest potential for causing infections as these species are the primary isolates from infected patients [65]. Some authors have suggested that the virulence and antibiotic resistance capability of some enterococci is even strain specific, considering the ecological niche they inhabit [55,72]. The statistical analyses of our results showed that the distribution of ABR genes is dependent on the ecological level but not on the species belonging. Our observations showed that the only multidrug-resistant strain was found in human breast milk. However, multidrug-resistant enterococci have also been reported in dairy products [67].

The virulence genes were much more present in the strains and a better generalization can be made. Significant differences were observed between strains from different origins as well as between different species (Figure 7). The greatest number of virulence genes was detected in *E. faecalis* strains from breast milk. The distribution of tested virulence-associated genes among the strains from the other samples was found to be species and even strain specific, because *E. faecalis* and *E. faecium* were not found in the snail GIT. Comparing the number of virulence genes distributed among *E. faecalis* and *E. faecium* isolates from food and human samples, we can conclude that this number drastically increased in the latter. All breast milk isolates carry genes for virulence factors, and four of them (*E. faecalis* BM5, BM6, BM9, and BM10) contain seven out of the eight screened virulence genes. Our results differ from those reported by Santana et al. [73], who investigated the distribution of *ace*, *efaA*, *gelE*, *cylA*, *hyl*, and *esp* virulence genes among an enterococcal population isolated from raw human breast milk. In their investigation, only two genes were detected (*efaA* and *ace*). We also noticed that two of our strains of *E. durans* (CM2 and CM3) isolated from cow milk did not carry any genes for virulence factors, as opposed to two strains of *E. durans* (YFC4 and YFC5) isolated from young feta cheese, which had the *cylB* gene.

Hemolytic activity is another virulence trait with great importance, as it enhances the severity of the caused infections. The production of cytolysin is associated with induced septicemia and a fivefold increased risk of acutely terminal outcome in patients [74]. In this study, none of the isolates had β-hemolytic activity, although some of the strains amplified the *cylB* gene. However, the ability of *E. faecalis* to express β-hemolysis has been reported [70]. It is known that the operon for cytolysin production is composed of five genes. The genes *cylL_l_* and *cylL_s_* encode the two structural subunits, which are then modified intracellularly by the product of the *cylM* gene. Then, they are transported out of the cell by a transporter encoded by the *cylB* gene. Once they are out of the cell, the precursor components are then activated by the *cylA* product. The gene *cylI* is responsible for the immunity of the bacteria to cytolysin. The regulation of expression is carried out by the products of two other genes—*cylR1* and *cylR2* [75,76,77,78]. In the present study, we established that the *cylB* gene did not correlate with the phenotypic hemolytic activity of the strains, which could be explained by an incomplete *cyl* operon. An interesting finding was that the highest percentage of α-hemolytic strains (45%) was established among species isolated from the GIT of *C. aspersum* (*E. durans*, *E. casseliflavus*, *E. gilvus*, *E. pseudoavium*, *E. pallens*, *E. malodoratus*, *E. devriesei*, and *E. gallinarum*). On the other hand, only one *E. faecalis* strain (BM4) isolated from human breast milk showed such activity. Moreover, α-hemolysis does not cause complete destruction of the red blood cells, which may limit the pathogenicity of the analyzed strains.

Gelatinase is an enzyme which is involved in the degradation of gelatin, collagen, casein, hemoglobin, etc. [79]. However, this feature of enterococcal isolates of non-hospital origin is poorly studied. For that reason, we examined the gelatinase phenotype and genotype in our collection. The expression of the *gelE* gene has been reported to be regulated by the products of different genes (*fsrA*, *fsrB*, and *fsrC*) in the *fsr* operon. Moreover, the expression of these genes has been described to be dependent on cell density [41]. Thus, the presence of *gelE* does not always produce a positive phenotype. Our results are in accordance with those reported from other authors [54,80,81]. Generally, our work demonstrates that *gelE* is present in 35% of our isolates, but only 4% were gelatinase producers (isolates from human breast milk). We can conclude that unexpressed *gelE* gene in most strains is due to one of the aforementioned reasons—lack of *fsr* operon or low cell density. Our observations indicate that the expression of the *gelE* gene and the manifestation of phenotypic gelatinase activity is a feature related to human isolates.

## 5. Conclusions

In this study, we tried to track the path of pathogenicity of potentially plant-associated enterococci in different levels of the alimentary chain. We established a step-by-step increase in the factors of virulence and ABR with maximal persistence in the human product—breast milk. This creates a serious problem and ambiguity—what will happen with these acquired pathogenic potential when these strains re-enter the environment and colonize the plant again? This study’s findings can be considered as a solid basis for future investigations.

## Figures and Tables

**Table 1:** Primer pairs used for detection of virulence and antibiotic resistance genes.

Primer	Sequence (5′ to 3′)	Tm (°C)	Product Size (bp)	Reference
Primers for virulence-related genes				
*cylB*-F	GGAGAATTAGTGTTTAGAGCG	57	522	[53]
*cylB*-R	GCTTCATAACCATTGTTACTATAGAAAC			
*esp*-F	CGATAAAGAGAGAGCGGAG	57	539	[53]
*esp*-R	GCAAACTCTACATCCACGTC			
*gls*24-F	GCATTAGATGAGATTGATGGTC	54	446	[53]
*gls*24-R	GCGAGGTTCAGTTTCTTC			
*psa*A-F	CTATTTTGCAGCAAGTGATG	54	540	[53]
*psa*A-R	CGCATAGTAACTATCACCATCTTG			
*agg*-F	AAGAAAAAGAAGTAGACCAAC	54	1553	[54]
*agg*-R	AAACGGCAAGACAAGTAAATA			
*ace*-F	AAAGTAGAATTAGATCACAC	51	320	[55]
*ace*-R	TCTATCACATTCGGTTGCG			
*gel*E-F	ACCCCGTATCATTGGTTT	51	419	[54]
*gel*E-R	ACGCATTGCTTTTCCATC			
*nucl*-F	GTGTAAAAGAAGTTACTGAAAATGTTACTC	62	332	[53]
*nucl*-R	GCGTTTTTTGTAGTAATGTTCCATCTACG			
Primers for antibiotic resistance-related genes				
*aac6′-aph2″*-F	CTGATGAGATAGTCTATGGTATGGATC	65	375	[53]
*aac6′-aph2″-*R	GCCACACTATCATAACCACTACCG			
*aph*A-F	GCCGATGTGGATTGCGAAAA	55	292	[56]
*aph*A-R	GCTTGATCCCCAGTAAGTCA			
*bla*Z-F	ACTTCAACACCTGCTGCTTTC	60	240	[57]
*bla*Z-R	TAGGTTCAGATTGGCCCTTAG			
*cat_pIP501_*-F	GGATATGAAATTTATCCCTC	50	486	[58]
*cat_pIP501_*-R	CAATCATCTACCCTATGAAT			
*gyr*A-F	ACTTGAAGATGTTTTAGGTGAT	55	559	[59]
*gyr*A-R	TTAGGAAATCTTGATGGCAA			
*erm*-F	CATTTAACGACGAAACTGGC	55	726	[59]
*erm*-R	GGAACATCTGTGGTATGGCG			
*erm*B-F	CATTTAACGACGAAACTGGC	52	405	[59]
*erm*B-R	GGAACATCTGTGGTATGGCG			
*mef*A-F	ACTATCATTAATCACTAGTGC	52	346	[60]
*mef*A-R	TTCTTCTGGTACTAAAAGTGG			
vanA36-F	TTGCTCAGAGGAGCATGACG	65	957	[61]
vanA992-R	TCGGGAAGTGCAATACCTGC			

**Table 2:** Enterococcal species identification.

No	Isolate	Origin	Species	Method of Identification	No.	Isolate	Origin	Species	Method of Identification
1	CA1	*C. aspersum*	*E. mundtii*	MALDI-TOF	37	BY8	Bulgarian yogurt	*Enterococcus* sp.	Sequencing
2	CA2	*C. aspersum*	*E. casseliflavus*	PCR, Sequencing	38	BY9	Bulgarian yogurt	*E. casseliflavus*	PCR, Sequencing
3	CA3	*C. aspersum*	*E. gilvus*	MALDI-TOF	39	BY10	Bulgarian yogurt	*E. faecalis*	PCR
4	CA4	*C. aspersum*	*E. mundtii*	MALDI-TOF	40	BY11	Bulgarian yogurt	*E. faecalis*	PCR
5	CA5	*C. aspersum*	*E. casseliflavus*	PCR, Sequencing	41	BY12	Bulgarian yogurt	*E. faecium*	PCR
6	CA6	*C. aspersum*	*E. mundtii*	Sequencing	42	BY13	Bulgarian yogurt	*E. faecium*	PCR
7	CA7	*C. aspersum*	*E. mundtii*	MALDI-TOF	43	BY14	Bulgarian yogurt	*E. faecium*	PCR
8	CA8	*C. aspersum*	*E. pseudoavium*	Sequencing	44	BY15	Bulgarian yogurt	*E. faecium*	PCR
9	CA9	*C. aspersum*	*E. pseudoavium*	Sequencing	45	BY16	Bulgarian yogurt	*E. faecium*	PCR
10	CA10	*C. aspersum*	*E. pallens*	Sequencing	46	BY17	Bulgarian yogurt	*E. gallinarum*	MALDI-TOF
11	CA11	*C. aspersum*	*E. malodoratus*	MALDI-TOF	47	BY18	Bulgarian yogurt	*E. casseliflavus*	PCR, Sequencing
12	CA12	*C. aspersum*	*E. casseliflavus*	PCR, Sequencing	48	BY19	Bulgarian yogurt	*E. casseliflavus*	PCR, Sequencing
13	CA13	*C. aspersum*	*E. devriesei*	Sequencing	49	BY20	Bulgarian yogurt	*E. casseliflavus*	PCR, Sequencing
14	CA14	*C. aspersum*	*E. gallinarum*	Sequencing	50	BY21	Bulgarian yogurt	*E. casseliflavus*	PCR, Sequencing
15	CA15	*C. aspersum*	*E. gallinarum*	Sequencing	51	BY22	Bulgarian yogurt	*E. faecalis*	PCR
16	CA16	*C. aspersum*	*E. devriesei*	MALDI-TOF	52	BY23	Bulgarian yogurt	*E. faecalis*	PCR
17	CA17	*C. aspersum*	*E. mundtii*	MALDI-TOF	53	BY24	Bulgarian yogurt	*E. faecalis*	PCR
18	CM1	Cow milk	*E. faecium*	PCR	54	BY25	Bulgarian yogurt	*E. faecalis*	PCR
19	CM2	Cow milk	*E. durans*	MALDI-TOF	55	BY26	Bulgarian yogurt	*E. faecalis*	PCR
20	CM3	Cow milk	*E. durans*	MALDI-TOF	56	BY27	Bulgarian yogurt	*E. faecalis*	PCR
21	CM4	Cow milk	*E. faecalis*	MALDI-TOF	57	BM1	Breast milk	*E. faecalis*	PCR
22	YFC1	Young feta cheese	*E. faecalis*	PCR	58	BM2	Breast milk	*E. faecalis*	PCR
23	YFC2	Young feta cheese	*E. durans*	PCR	59	BM3	Breast milk	*E. faecalis*	PCR
24	YFC3	Young feta cheese	*E. faecalis*	PCR	60	BM4	Breast milk	*E. faecalis*	PCR
25	YFC4	Young feta cheese	*E. durans*	PCR	61	BM5	Breast milk	*E. faecalis*	PCR
26	YFC5	Young feta cheese	*E. durans*	PCR	62	BM6	Breast milk	*E. faecalis*	PCR
27	MFC1	Matured feta cheese	*E. faecium*	PCR	63	BM7	Breast milk	*E. faecalis*	PCR
28	MFC2	Matured feta cheese	*E. faecium*	PCR	64	BM8	Breast milk	*E. faecalis*	PCR
29	DK1	Doner kebab	*E. faecium*	PCR	65	BM9	Breast milk	*E. faecalis*	PCR
30	BY1	Bulgarian yogurt	*E. faecium*	MALDI-TOF	66	BM10	Breast milk	*E. faecalis*	PCR
31	BY2	Bulgarian yogurt	*E. faecalis*	PCR	67	BM11	Breast milk	*E. faecalis*	PCR
32	BY3	Bulgarian yogurt	*E. faecalis*	PCR	68	BM12	Breast milk	*E. faecalis*	PCR
33	BY4	Bulgarian yogurt	*E. faecalis*	PCR	69	BM13	Breast milk	*E. faecalis*	PCR
34	BY5	Bulgarian yogurt	*E. faecalis*	PCR	70	BM14	Breast milk	*E. faecalis*	PCR
35	BY6	Bulgarian yogurt	*E. faecalis*	PCR	71	BM15	Breast milk	*E. faecalis*	PCR
36	BY7	Bulgarian yogurt	*Enterococcus* sp.	Sequencing	72	BM16	Breast milk	*E. faecalis*	PCR

**Table 3:** Distribution of phenotypic antibiotic resistance among the enterococcal isolates.

ABR Phenotype	Number of Isolates	Species Identification	Origin of Isolation
One Antibiotic			
AMP	12	*E. faecium DK1*	Doner kebab
		*E. gallinarum BY17*	Bulgarian yogurt
		*E. mundtii CA1*	*C. aspersum*
		*E. malodoratus CA11*	
		*E. devriesei CA13*	
		*E. faecalis BM3*	Human breast milk
		*E. faecalis BM4*	
		*E. faecalis BM5*	
		*E. faecalis BM6*	
		*E. faecalis BM9*	
		*E. faecalis BM12*	
		*E. faecalis BM14*	
Two antibiotics			
AMP + ERV	1	*E. faecium CM1*	Cow milk
AMP + TG	1	*E. faecalis YFC1*	Young feta cheese
GEN + RP	1	*E. faecalis BM7*	Human breast milk
Three antibiotics			
AMP + HLS + RP	1	*E. faecalis* BM15	Human breast milk

**Table 4:** Distribution of genes encoding virulence factors among the tested enterococcal population.

Strains	Virulence Genes								Strains	Virulence Genes							
	*cyl B*	*esp*	*gls 24*	*nucl*	*psa*	*agg*	*gel E*	*ace*		*cyl B*	*esp*	*gls 24*	*nucl*	*psa*	*agg*	*gel E*	*ace*
*E. faecium* CM1									*E. faecalis* BY25								
*E. durans* CM2									*E. faecalis* BY26								
*E. durans* CM3									*E. faecalis* BY27								
*E. faecalis* CM4									*E. mundtii* CA1								
*E. faecalis* YFC1									*E. casseliflavus* CA2								
*E. durans* YFC2									*E. gilvus* CA3								
*E. faecalis* YFC3									*E. mundtii* CA4								
*E. durans* YFC4									*E. casseliflavus* CA5								
*E. durans* YFC5									*E. mundtii* CA6								
*E. faecium* MFC1									*E. mundtii* CA7								
*E. faecium* MFC2									*E. pseudoavium* CA8								
*E. faecium* DK1									*E. pseudoavium* CA9								
*E. faecium* BY1									*E. pallens* CA10								
*E. faecalis* BY2									*E. maloduratus C*A11								
*E. faecalis* BY3									*E. casseliflavus* CA12								
*E. faecalis* BY4									*E. devriesei* CA13								
*E.faecalis* BY5									*E. gallinarum* CA14								
*E.faecalis* BY6									*E. gallinarum* CA15								
*E. species* BY7									*E. devriesei* CA16								
*E. species* BY8									*E. mundtii* CA17								
*E. casseliflavus* BY9									*E. faecalis* BM1								
*E. faecalis* BY10									*E. faecalis* BM2								
*E. faecalis* BY11									*E. faecalis* BM3								
*E. faecium* BY12									*E. faecalis* BM4								
*E. faecium* BY13									*E. faecalis* BM5								
*E. faecium* BY14									*E. faecalis* BM6								
*E. faecium* BY15									*E. faecalis* BM7								
*E. faecium* BY16									*E. faecalis* BM8								
*E. gallinarum* BY17									*E. faecalis* BM9								
*E. casseliflavus* BY18									*E. faecalis* BM10								
*E. casseliflavus* BY19									*E. faecalis* BM11								
*E. casseliflavus* BY20									*E. faecalis* BM12								
*E. casseliflavus* BY21									*E. faecalis* BM13								
*E. faecalis* BY22									*E. faecalis* BM14								
*E. faecalis* BY23									*E. faecalis* BM15								
*E. faecalis* BY24									*E. faecalis* BM16								

Pink color—negative result, no amplification product; green color—positive result—specific amplification product.

## Data Availability

The data presented in this research are available in the manuscript.
